# Mitral stenosis with term pregnancy: how to manage this case?

**DOI:** 10.11604/pamj.2013.14.144.2439

**Published:** 2013-04-10

**Authors:** Zine el abidine Benali, Houssam Ahmaidi, Karim Rachidi, Driss Omari

**Affiliations:** 1 Department of Anesthesiology, CHP Eddarak, Berkane, Morocco; 2 Department of maternity, CHP Eddarak, Berkane, Morocco; 3 Department of Internal Medicine and Cardiovascular Diseases, CHP Eddarak, Berkane, Morocco

**Keywords:** Mitral stenosis, pregnancy, heart disease, rheumatic heart disease

## Abstract

From heart disease complicating pregnancy, rheumatic mitral stenosis occupies a larger part. Physiological changes during pregnancy and the impact of pathological mitral stenosis on pregnancy and labor are important to know. A multidisciplinary approach to the diagnosis and treatment reduces mortality and morbidity. We report the case of a patient with mitral stenosis moderately tight with term pregnancy and perioperative management.

## Introduction

Pregnancy is a high risk period for women with mitral stenosis (MS). Women of childbearing age met the MS is almost always rheumatic origin. The maternal and perinatal complications during pregnancy in women with unfavorable reflect the interaction between the normal cardiovascular changes of pregnancy and the narrowed mitral valve. We report the case of a pregnant woman at 38 weeks gestation followed wrong with MS moderately tight, admitted intensive care unit.

## Patient and observation

**Figure 1:**
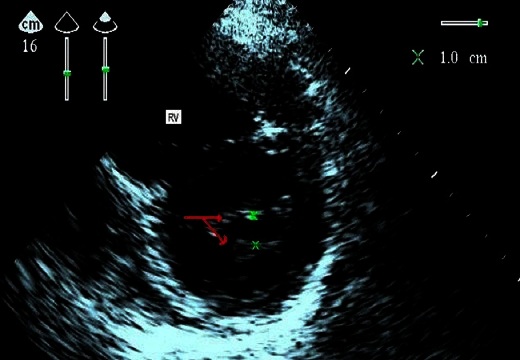
Parasternal short-axis 2D echocardiographic image in diastole showing thickening and stenosis of the mitral valve. (RV: right ventricle, arrows: thickening of the large and small mitral valve)

**Figure 2:**
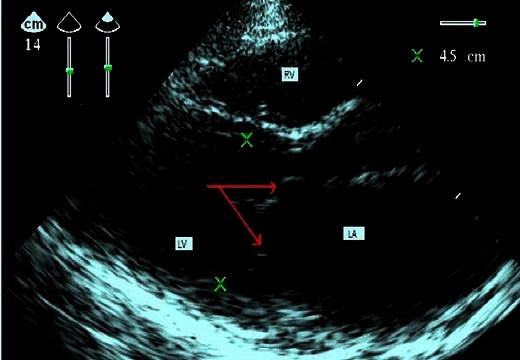
Parasternal long-axis 2D echocardiographic image in diastole showing the incomplete opening of the mitral valve. (LA: left Atrium, LV: left ventricle, RV: right ventricle, arrows: thickening of the large and small mitral valve)

A woman aged 26 years, nulliparous, pregnant 38 weeks gestation, with small size 1 meter 50 and a tight mitral stenosis. In this background, we noted repeated episodes of angina, and poor oral health. At admission, she was hemodynamically stable with a blood pressure 120/80 mm Hg, weight 60 kg, had a shallow tachypnea to 23 cycles / minute. On examination, she had dyspnea NYHA Stage II (Classification of the New York Heart Association), bilateral edema of the lower limbs, cardiopulmonary auscultation revealed a rolling end diastolic mitral with intensity 3/6, a burst of B2, tachycardia, and crackles at two discrete lung bases, no evidence for an infringement of the right cavities were observed. The electrocardiogram betrayed bifid P waves, wide (> 0.11 sec) in DI, DII and aVL, in favor of an enlarged left atrium. Echocardiography showed a mitral stenosis moderately tight with an area of 1.3cm^2^ cut short axis planimetry, valvular thickening with the large and small mitral valve (Figure 1, Figure 2), left atrial dilatation with a volume of 32 ml/m^2^ without spontaneous contrast or thrombus detected, moderate pulmonary arterial hypertension, global and segmental contractility (17 segments) to the limit of normal with an ejection fraction 53% left ventricular, pericardium dry, and vena cava inferior to 13 cm in diameter with normal respiratory variation. The results of standard laboratory tests were normal. On the obstetric examination is for a narrows pelvis, after a multidisciplinary discussion cesarean section under spinal anesthesia was determined.

Premedication consisted of cimétidine 200 mg, hydroxyzine, antibiotic associated with the imidazole and Beta lactam one hour before induction of anesthesia. The patient has never previously received drugs under heart. Throughout the procedure, blood pressure, oxygen saturation, heart rate, urine output, and ECG were monitored. Spinal anesthesia with 10 mg of isobaric bupivacaine 0.5% with one injection speed very slow under 3 min and nasal oxygen 4 liter/min. Incidents encountered perioperatively were hypotensive episodes, tachycardia and amounted to 96/min, these incidents were just after the installation of the engine block of the two lower limbs were readily reversible after injection titration of ephedrine, extraction of a male baby APGAR score: 10/10 with a weight of 3 kg. The venous thromboembolic disease prevention was established at the tenth hour postoperative: enoxaparin 0.4 ml subcutaneously, multimodal analgesia, diuretics, filling echo guided, and digitalis. The patient transferred on the fourth day in the maternity ward with cardiac monitoring.

## Discussion

The mitral orifice normally is 4 to 5cm^2^ area by planimetry in mode 2 D echocardiography short axis, with little or no MS tight: >1.5cm^2^, MS moderately tight: 1 to 1.5cm^2^, MS tight: <1cm^2^. In our patient we found an area of 1.3cm ^2^ with a MS moderately tight showing the severity of this disease in association with pregnancy, The MS encountered in women of childbearing age is almost always rheumatic origin. The maternal and perinatal complications during pregnancy in women with MS reflect the unfavorable interaction between the normal cardiovascular changes of pregnancy and MS.

In MS, the valve limit diastolic filling of the left ventricle, which results in a high gradient transmitral and left atrial pressure, and which are further increased by the hyper blood volume and physiological tachycardia during pregnancy, which increases the risk of pulmonary edema. This risk persists and increases during labor and delivery due to uterine contraction, and in connection with the venous return of the lower limbs when the uterus is emptied. The sudden increase in pre-load immediately after delivery, auto transfusion due to the uterus, can flood the central circulation, resulting in a severe pulmonary edema. In addition, there continues to be autologous blood for 24-72 hours after delivery. Thus, this risk extends over several days after birth [1]. Hence the interest of diuretics and filling echo guidance as in this case clinical. The hyper coagulability associated with pregnancy, when combined with the increase in left atrial pressure increases the risk of atrial fibrillation and thrombus formation. Hence the rapid establishment of anticoagulants and antiarrhythmic postoperatively. If MS is diagnosed before pregnancy, mitral commissurotomy is preferable if the valve morphology is acceptable. During pregnancy, the second trimester is the preferred time for any invasive procedure. Valvuloplasty using the technique of balloon became the accepted treatment for patients with symptomatic MS, and the success rate of close to 100% [2].

The objectives of the anesthetic management of patients with MS are: maintaining an acceptable slow heartbeat, immediate treatment of acute atrial fibrillation and a return to sinus rhythm, avoidance of aorto-caval compression, maintenance of adequate venous return, the maintenance of normal blood pressure and preventing pain before it starts, but not forgotten to fight against hypoxia, hypercapnia and acidosis. Antibiotic prophylaxis of endocarditis is reserved only for patients with a history of endocarditis or the presence of established infection [3]. There are no studies examining the best type of anesthetic technique in these patients. General anesthesia has the disadvantage of increased pulmonary arterial pressure and tachycardia during endotracheal intubation. In addition, the harmful effects of positive pressure ventilation on venous return may ultimately lead to heart failure [4]. Regional anesthesia has proven to be a safe technique in cardiac patients with a cesarean section [5], as in our patient. Whatever the mode of delivery and anesthesia technique, these patients are at high risk of hemodynamic stress due to auto transfusion of blood from the uterus. This can lead to pulmonary hypertension, pulmonary edema and congestive heart failure. Therefore, intensive monitoring and treatment should be continued until hemodynamic parameters return to normal with an extension for several days after surgery in intensive care unit. The maternal prognosis seems to correlate with the NYHA functional classification and incidence of maternal cardiac complications is closely related with the severity of MS [6, 7].

## Conclusion

An understanding the physiological changes of pregnancy, mitral stenosis with pathological consequences during the critical period in pregnant women, and especially an adequate perioperative management multidisciplinary between the anesthesiologist cardiologist and obstetrician, converge towards a reduction in mortality and morbidity peripartum.
